# Comparison of Conjugates Obtained Using DMSO and DMF as Solvents in the Production of Polyclonal Antibodies and ELISA Development: A Case Study on Bisphenol A

**DOI:** 10.3390/antib13040089

**Published:** 2024-10-29

**Authors:** Anna N. Berlina, Nadezhda S. Komova, Kseniya V. Serebrennikova, Anatoly V. Zherdev, Boris B. Dzantiev

**Affiliations:** A.N. Bach Institute of Biochemistry, Research Center of Biotechnology of the Russian Academy of Sciences, Leninsky Prospect 33, 119071 Moscow, Russia; nad4883@yandex.ru (N.S.K.); ksenijasereb@mail.ru (K.V.S.); zherdev@inbi.ras.ru (A.V.Z.); dzantiev@inbi.ras.ru (B.B.D.)

**Keywords:** bisphenol A, BVA, polyclonal antibody, organic solvent, dimethylformamide, dimethyl sulfoxide, hapten–protein conjugate

## Abstract

When developing immunochemical test systems, it is necessary to obtain specific antibodies. Their quality depends, among other things, on the immunogen used. When preparing hapten–protein conjugates to obtain antibodies for low-molecular-weight compounds, the key factors are the structure of the hapten itself, the presence of a spacer, the size of the carrier protein and the degree of its modification by hapten molecules. This work shows that one additional factor—the conditions for obtaining the hapten–protein conjugate—is overlooked. In this work, we have synthesized conjugates of bisphenol A derivative 4,4-bis(hydroxyphenyl)valeric acid (BVA), the protein carrier soybean trypsin inhibitor (STI), and bovine serum albumin (BSA) in reaction media combining water with two organic solvents: dimethylformamide (DMF) or dimethyl sulfoxide (DMSO). Namely, BSA_DMF_–BVA, STI_DMF_–BVA, BSA_DMSO_–BVA and STI_DMSO_–BVA conjugates were obtained. Rabbit polyclonal antibodies against the BSA_DMF_–BVA conjugate demonstrated basically different interactions in the developed ELISA systems using either STI_DMF_–BVA or STI_DMSO_–BVA conjugates. The use of the STI_DMF_–BVA conjugate demonstrated the absence of competition in combination with antisera obtained from BSA_DMF_–BVA in an ELISA. A competitive interaction was observed only with the use of the STI_DMSO_–BVA conjugate. Under the selected conditions, the detection limit of bisphenol A was 8.3 ng/mL, and the working range of determined concentrations was 18.5–290.3 ng/mL. The obtained data demonstrate the possibility of achieving sensitive immunoassays by simply varying the reaction media for the hapten–protein conjugation, which could provide an additional tool in the development of immunoassays for other low-molecular-weight compounds.

## 1. Introduction

Developing specific antibodies to low-molecular-weight substances has long become a routine immunization procedure; although, it is impossible to predict the immune response of a laboratory animal in advance [1]. Only the result of competitive immunoassay can confirm the quality and affinity of tested antibodies.

It is known that low-molecular-weight compounds whose molecular weight does not exceed 3 kDa are not capable of independently inducing an immune response since, from the point of view of immune recognition, they are incomplete antigens or haptens [2,3,4,5,6]. Therefore, the hapten molecule is enlarged with a carrier protein by synthesizing hapten–protein conjugates, thereby converting the hapten into a full-fledged antigen [7,8,9]. There are various methods for synthesizing hapten–protein conjugates, but traditionally, the latter should have amino or carboxyl groups available for modification. Since the carrier protein molecule is significantly larger than the hapten, it is expected that most of the antibodies are directed toward recognizing the carrier protein in the antiserum during immunization. However, a small portion of polyclonal antibodies directed to the hapten is sufficient for the development of immunoassay schemes for the determination of a low-molecular analyte.

Bisphenol A is a low-molecular-weight compound belonging to a group of bisphenols that is widely used in the production of packaging materials and plastic coatings [10]. This compound is regulated in food products and drinking water since it has an estrogen-like effect due to the fragmentary similarity of chemical structures [11,12,13]. To obtain hapten–protein conjugates, its derivatives containing a carboxyl group are usually used to form covalent bonds with an amine group of proteins. In the case of developing antibodies to bisphenol A, either a derivative—4,4′-bis(4-hydroxyphenyl)valeric acid (BVA) [14,15]—or compounds obtained by chemical synthesis with a spacer and a carboxyl group at the end are used [16].

A study of the literature on this topic allowed us to identify the main trends. Firstly, the main focus is typically on the modification of the hapten to obtain the immunogen derivative. Secondly, almost no researchers attach importance to the method of the synthesis of the hapten–protein conjugate and use “nomadic” synthesis protocols when referring to them. Thirdly, due attention is not paid to other components of the synthesis, for example, the use of an organic solvent in an aqueous environment, and its effect on the final results are not studied. In this work, we decided to demonstrate how the choice of solvent can influence the specificity of recognition of the target analyte, using the resulting antibodies to bisphenol A as an example. For this purpose, several conjugates were synthesized and compared in ELISA.

## 2. Materials and Methods

### 2.1. Materials

Bisphenol A (BPA), 4,4-bis(hydroxyphenyl)valeric acid (BVA), Tween-80, bovine serum albumin (BSA), soybean trypsin inhibitor (STI), dimethylsulfoxide, Tween-80, N-hydroxysulfosuccinimide (sulfo-NHS), and 1-ethyl-3-(3-dimethylaminopropyl)carbodiimide (EDC) were sourced from Sigma-Aldrich (St. Louis, MO, USA). Sheep polyclonal antibodies against rabbit IgG labeled with horseradish peroxidase (SARI-HRP) were from IMTEK (Moscow, Russia). Liquid colorimetric substrate 3,3′,5,5′-tetramethylbenzidine (TMB) with H_2_O_2_ (substrate solution) was obtained from Immunotech (Moscow, Russia). Methanol was from Fluka (Buchs, Switzerland). Buffers and water solutions were prepared with the use of purified water with a resistance of not more than 18.6 MΩ·cm at 25 °C (Simplicity Water Purification System, Millipore, Bedford, MA, USA). Complete and incomplete Freud’s adjuvants were from ServiceBio (Wuhan, China).

Transparent 96-well polystyrene microplates were from Costar (Corning Costar, MO, USA). Syringe filters of 0.22 µm for purifying samples and filtration of water solutions were from Sartorius (Gettingen, Germany).

Furthermore, 10 mM phosphate-buffered saline, pH 7.4 (PBS), was prepared by dissolving PBS tablets (EKO service, Saint-Petersburg, Russia) and used as a buffer for hapten–protein adsorption. A 10 mM phosphate-buffered saline, pH 7.4, with 0.05% Tween-80 (PBST, pH 7.4) was used as a washing buffer in our study.

### 2.2. Synthesis of the Conjugates of Hapten and Carrier Protein

The synthesis of hapten and conjugates was provided by the method of activated esters with the use of two types of organic solvents—DMF (BSA_DMF_–BVA and STI_DMF_–BVA) and DMSO (BSA_DMSO_–BVA and STI_DMSO_–BVA). The initial protocol of synthesis was taken from [14,15]. The concentrations of both DMF and DMSO solvents were the same during the synthesis. The amounts of substances were proportionally reduced for the second synthesis.

For the synthesis of BSA_DMF_–BVA and STI_DMF_–BVA, 6.1 mg of NHS and 10.4 mg of EDC were dissolved in 400 µL of Milli-Q water, and then, 12.4 mg of BVA in 400 µL of DMF was added. The resulting mixture was shaken for 24 h. A total of 25.4 mg of a carrier protein (STI or BSA) was dissolved in 1 mL of 10 mM PBS, pH 7.4, and added to the first mixture. The mixture was shaken for 24 h, and then, conjugates were dialyzed for 3 days against 10 mM PBS with 2× buffer exchange.

To obtain BSA_DMSO_–BVA and STI_DMSO_–BVA, 2.4 mg of NHS and 4.09 mg of EDC were dissolved in 200 µL of Milli-Q water. In total, 4.88 mg of BVA was dissolved in 200 µL of DMSO and mixed with the solution of activators. The resulting mixture was vortexed for 24 h. A total of 10 mg of a carrier protein (STI or BSA) was dissolved in 1 mL of 10 mM PBS, pH 7.4, and added to the first mixture. The mixture was shaken for 5 h, and then, the resulting conjugates were dialyzed against 10 mM PBS with a 2× buffer exchange.

All conjugate preparations were filtered through 0.22 µm membrane filters to remove aggregates after dialysis and prior to use. The concentration of preparations was calculated from the spectra of adsorption and absorbance at 280 nm. The conjugates were aliquoted and stored at −20 °C until use.

### 2.3. Characteristics of the BVA–Protein Conjugates

The resulting conjugates were characterized by UV-vis spectrophotometry and FT-IR. Biochrom Libra S80 spectrophotometer (Biochrom, Cambridge, UK) was used to obtain the spectra of adsorption in the wavelength range of 220–700 nm.

To provide FT-IR investigations, the conjugates were preliminarily lyophilized with the use of a Martin Christ freeze dryer (Alpha 1–2 LD Plus, Osterode, Germany). The Fourier transform infrared (FT-IR) spectra of all preparations, including pure proteins and native haptens, were obtained in the wave range between 4000 and 500 cm^−1^ using an FT/IR-6700 spectrophotometer (JASCO, Tokyo, Japan).

### 2.4. Circular Dichroism Measurements

Circular dichroism (CD) spectra were recorded using the CD spectrometer Chirascan from Applied Photophysics (Leatherhead, UK) [17]. The CD spectra of native proteins (BSA and STI), corresponding to conjugates with and without of DMF and DMSO, were recorded at 20 °C. All the solutions were prepared in 10 mM PBS at a 1 mg/mL concentration of protein/conjugate. To avoid pre-aggregation, all protein and conjugate solutions were filtered through a filter with a pore diameter of 0.22 µm. The CD measurements were collected in the range of 190–280 nm in the 0.1 mm quartz cuvette.

### 2.5. Rabbit Immunization and Obtaining of Polyclonal Antibodies

Immunization of rabbits was carried out according to the previously described protocol [18].

All animal experiments complied with the legislation of the Russian Federation and were approved by the institutional ethics committee for animal experiments. The animals were handled in accordance with the European Convention for the Protection of Vertebrate Animals Used for Experimental and Other Scientific Purposes (Directive 2010/63/EU of 1 January 2013). A gray chinchilla rabbit (female, weighing 3 kg), obtained from the breeder of laboratory animals “Manikhino” (Moscow region, Russia), was immunized with a hapten–protein conjugate. The first immunization solution was prepared as follows: the immunogen (BSA_DMF_–BVA) was dissolved in 0.9% saline and emulsified with an equal volume of Freund’s complete adjuvant to a final concentration of 1.0 mg/mL. The resulting emulsion with a volume of 1.3 mL was injected subcutaneously into the posterior part of the animal’s body in six places. After this, the rabbit was revaccinated with a half dose according to the following scheme: days 14/56 in 0.9% saline with incomplete Freund’s adjuvant (1:1) subcutaneously and days 28/42 at 0.9% saline solution with incomplete Freund’s adjuvant *v*/*v* (1:1) intramuscularly. Blood was collected one week after dosing (days 35/49/63, antisera №1 and №2 were obtained on the 63rd day) from the ear vein. Immunization of BSA_DMSO_–BVA rabbits was carried out by the same technique. Blood samples were allowed to settle for 1 h at +4 °C. The serum was separated by centrifugation for 15 min at 4000× *g* and +4 °C and stored at −20 °C.

### 2.6. ELISA Optimization

To optimize the assay, the following parameters were selected and varied. The conjugate for the immobilization of STI–BVA was adsorbed into the wells of the plate at concentrations of 0.5, 1.0, 2.0 and 3.0 μg/mL. To select the optimal dilution of the antiserum for use in the competitive assay, the antisera were titrated in various dilutions from 1:1000 to 1:10,000,000. The selection criterion was the antiserum dilution giving an optical density value of 1.0. Additionally, to assess the reproducibility and detection limits of bisphenol A, a competitive interaction was carried out at the selected antiserum dilutions of 1:10,000, and 1:25,000, and bisphenol A concentrations varied from 0.19 to 11,000 ng/mL.

### 2.7. Colorimetric ELISA

Rabbit sera were tested using non-competitive and competitive enzyme-linked immunosorbent assay formats. First, 1 μg/mL STI–BVA conjugate in 10 mM PBS was immobilized into the wells of a transparent microplate, followed by overnight incubation. The microplates were washed three times with PBST and then incubated at 37 °C for 1 h with rabbit antiserum (rAs) diluted between 1:100 and 1:102,400. The manifestation of immune complexes was ensured by incubation of the microplate at +37 °C with goat anti-rabbit IgG labeled with horseradish peroxidase (GARI–HRP) at a dilution of 1:5000 in PBST. Next, 100 μL of substrate solution (TMB + H_2_O_2_, Immunotech, Russia) was added to the wells of the microplate. Finally, the reaction was stopped with 50 μL of 0.1 M H_2_SO_4_.

To perform a competitive enzyme immunoassay, 1 μg/mL of STI–BVA in 50 mM PBS was adsorbed into the microplate wells. After washing three times, 50 μL of BPA in a concentration range of 3000–0.01 ng/mL and 50 μL of rAs in the selected dilution were added, followed by incubation for 1 h at +37 °C. Subsequent stages of analysis were similar to those described above.

The samples were analyzed using bisphenol A-free drinking water samples with known analyte concentrations added. The prepared fortified samples were introduced at the competitive interaction stage. In this case, the antiserum solution in PBST containing 0.1% Tween-80 was added since the samples were not pre-diluted with buffer. The sample-to-antiserum solution volume ratio was 1:1.

### 2.8. ELISA Data Processing

Based on the measurement results, the dependence of the optical density at 450 nm on the concentration of the target compound was plotted using Origin 9.0 software (OriginLab Corporation, Northampton, MA, USA). To process the results of non-competitive ELISA, the dependence of optical density at 450 nm on antisera dilution was plotted. To perform a competitive ELISA, the antiserum dilution was selected where the OD_450_ was approximately 1.0. The competitive dependencies of OD_450_ on BPA concentration were approximated by a four-parameter sigmoidal equation to obtain a calibration curve.

### 2.9. Determination of Free Amino Groups in the Conjugates

Free amino groups in the conjugates and native proteins were determined using fluorescamine, as in the protocol in [19]. For this purpose, 10 mg of fluorescamine was dissolved in 150 μL of acetone. Hapten–protein conjugates and native proteins were taken for testing at a concentration of 20 μg/mL in 10 mM PBS in a volume of 200 μL. A total of 10 μL of fluorescamine solution was added to each solution and kept for 5 min at RT under shaking using an Intellimixer (ELMI, Riga, Latvia) to obtain the labeled protein. After this reaction, 150 μL of each solution was transferred to the wells of white microplates (from Nunc, Roskilde, Denmark) for fluorescence measurements. They were performed at an excitation wavelength of 390 nm in the range of 450–550 nm using an EnSpire 2300 microplate reader from Perkin Elmer (Waltham, MA, USA). The calculation of the fluorescence loss has been carried out at the emission maximum of 490 nm (from the spectra) for all preparations.

## 3. Results and Discussion

### 3.1. Preparation and Characterization of Hapten–Protein Conjugates

**Figure 1:**
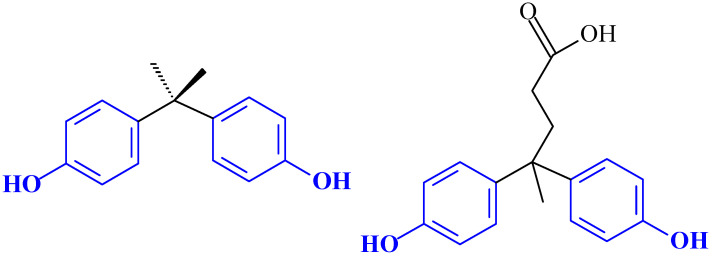
Structures of BPA and 4,4-bis(hydroxyphenyl)valeric acid (BVA).

The development of specific antibodies and developing an immune response in the animal’s body requires the introduction of an antigen into the animal’s body. Low-molecular-weight compounds are not capable of directly inducing an immune response, which requires artificial enlargement of the incomplete antigen (hapten) by a carrier protein molecule for further immunization. Since the structure of bisphenol A does not have functional groups available for direct conjugation to carrier proteins, conjugates for immunization of rabbits as laboratory animals were prepared using the bisphenol A derivative 4,4-bis(hydroxyphenyl)valeric acid (BVA) (Figure 1). This compound provides steric accessibility to two aromatic rings containing phenolic hydroxyls without direct modification.

Since the structure of BVA contains the desired carboxyl group, the preparation of hapten–protein conjugates was carried out using the activated ester method, in which the carboxy group of BVA is activated and subsequently interacts with the amino groups of the carrier protein. In this study, BSA and STI, which are commonly used in biomolecular systems, were chosen as carrier proteins due to their stability, non-glycosylated nature and ease of modification. In addition, BSA has about 30 primary surface amino groups in its structure available for modification [20,21]. As for STI, its structure only contains ten amino groups [22], the modification of which will allow for the development of a drug with a small hapten load on its surface, suitable for competitive analysis.

The preparation of activated BVA derivatives and conjugation with proteins was carried out in the presence of two solvents—DMF and DMSO. Thus, four conjugates were obtained—BSA_DMF_–BVA, STI_DMF_–BVA, BSA_DMSO_–BVA and STI_DMSO_–BVA. The BSA_DMF_–BVA conjugate was used to immunize rabbits to obtain specific antibodies.

When synthesizing the BSA_DMF_–BVA and STI_DMF_–BVA conjugates, pronounced aggregation of the protein component was observed during the reaction between the amino groups of the protein and the activated carboxyls of the hapten, which subsequently required purification of the conjugate from aggregates. The measurement of optical density at 280 nm was carried out in purified preparations. Therefore, the next step in the work was an attempt to change the method of synthesis of the protein–BVA conjugate, replacing the organic solvent DMF with DMSO and reducing its content. It is known that the latter has a lesser effect on protein aggregation in an aqueous environment. Thus, in reference [23], it was shown that the aggregation ability of DMF is higher than that of DMSO. In the presence of varying amounts of organic solvents, changes in protein conformation and compaction are possible [24].

**Figure 2:**
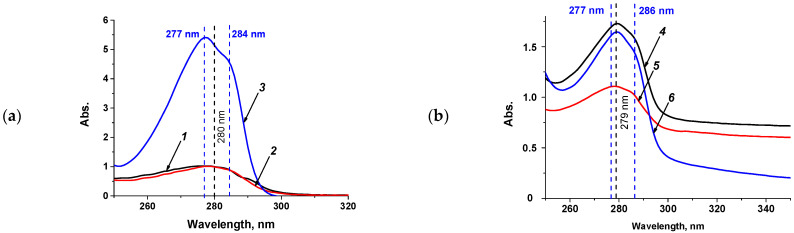
Spectra of native proteins, BVA (**a**), and conjugates (**b**). (*1*)—STI (1.0 mg/mL), (*2*)—BSA (1.0 mg/mL), (*3*)—BVA (1.0 mg/mL). The absorption spectra of the preparations were measured using a 10 mm thick cuvette. (*4*)—BSA_DMSO_–BVA (13 mg/mL), (*5*)—BSA_DMF_–BVA (12.66 mg/mL), (*6*)—STI_DMSO_–BVA (8.3 mg/mL). The absorption spectra of the preparations were measured using a 1 mm thick cuvette.

The resulting conjugates were characterized by UV-vis spectrometry and FT-IR. The study of absorption spectra in a wide range of wavelengths revealed the presence of two main peaks—at 278–280 nm for both conjugates and native proteins, and a small “shoulder” at 286 nm—for all conjugate preparations (Figure 2). Native BVA has a main peak at 277 nm and a shoulder at 284 nm. Conjugates with BSA have a main absorbance peak at 279 nm and a shoulder at 286 nm, and conjugates with STI have a main absorbance peak at 278 nm and a shoulder at 285 nm, respectively. The absorption spectra indicate the successful synthesis of hapten–protein conjugates.

**Figure 3:**
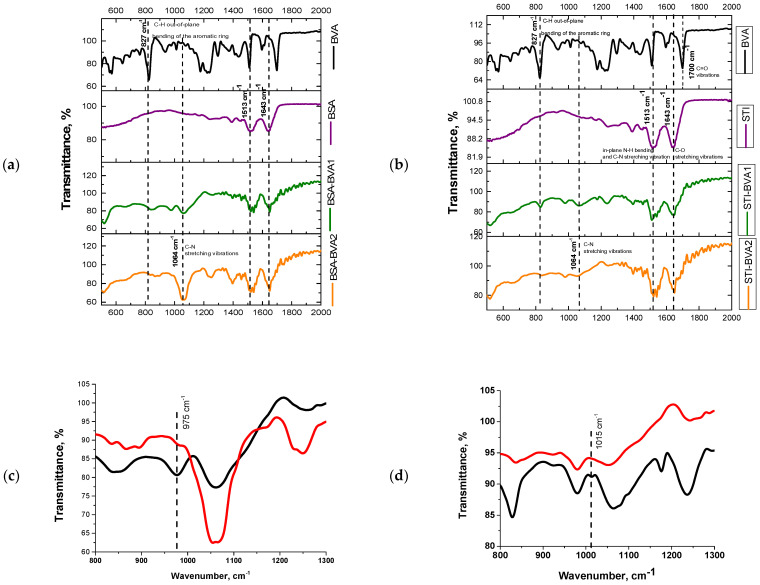
Characterization of BVA, conjugates and native proteins by FT-IR technique. (**a**) BVA, BSA, BSA_DMF_–BVA and BSA_DMSO_–BVA; (**b**) BVA, STI, STI_DMF_–BVA, STI_DMSO_–BVA; (**c**) BSA_DMF_–BVA (black line) and BSA_DMSO_–BVA (red line) at wavenumber range of 800–1300 cm^−1^; (**d**) STI_DMF_–BVA (black line) and STI_DMSO_–BVA (red line) at wavenumber range of 800–1300 cm^−1^.

Comparison of FT-IR spectra data showed similarities and differences in stretching vibrations of both the original protein and hapten preparations and the conjugates. Thus, the presence of a free carboxyl group and, accordingly, characteristic C=O stretching vibration at 1700 cm^−1^ and C-H bonds in carbon moiety at 827 cm^−1^ are observed for BVA (Figure 3a,b). It is known that vibrations in the range of 800–1000 cm^−1^ are characteristic of C-C bonds in alkanes, which is typical for a carbon spacer in a BVA radical containing a carboxyl group [25]. When BVA is conjugated with carrier proteins, the peak at 1700 cm^−1^, characteristic of the C=O bond, disappears (Figure 3a,b). The stretching vibration at 1600 cm^−1^ specific for sp^2^ hybridized carbon is observed for both native proteins and conjugates, which is logical if both the protein structure and a large number of aromatic rings in the structures of the amino acids that make up the protein are preserved.

The stretching vibrations at 1513–1632 cm^−1^, 1230–1300 cm^−1^ and 1064 cm^−1^ are characteristic for amide bonds in conjugates composition [26,27]. These vibrations are absent in native hapten preparations. Almost-identical bands in the region of 2800–3000 cm^−1^ for all four conjugates are a sign of asymmetric and symmetric stretching vibrations of C-H [28], which is typical for the alkyl region.

The presence of key peaks indicates the successful synthesis of hapten–protein conjugates using the activated ester method. Moreover, if you look at the spectra of the conjugates in the selected ranges (800–1300 cm^−1^), they are quite different depending on the chosen solvent and carrier protein (Figure 3c—for BSA, Figure 3d—for STI). The band strengths of 975 cm^−1^ in BSA_DMF_–BVA and 1015 cm^−1^ in STI_DMF_–BVA, which are absent in native proteins and conjugates BSA_DMSO_–BVA and STI_DMSO_–BVA, were attributed to C-C and C-N, similar to the work in [26]. Despite the fact that, in preparing this work, the conjugates were obtained without the use of formaldehyde for immunizing animals, the presence of such vibrations is indicated by the intermediate products formed.

### 3.2. Circular Dichroism Spectroscopy

Using the circular dichroism method, it is possible to evaluate the initial secondary structure of proteins, as well as to evaluate its changes during the interaction of proteins with various substances in solution and reaction mixture [29,30,31,32]. When analyzing proteins using the CD method, the main peaks at 209 and 220 nm for α-helix conformation and 218 nm observed for β-sheets are important [33].

**Figure 4:**
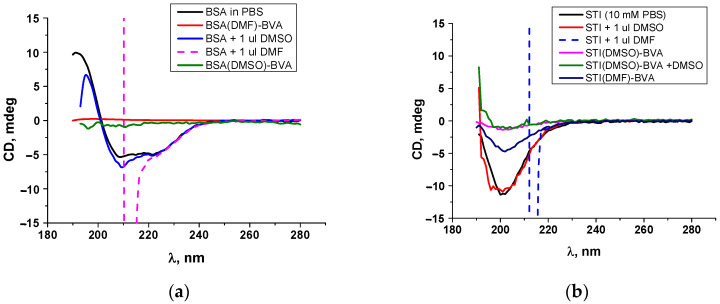
Characterization of native proteins and corresponding conjugates by circular dichroism spectroscopy. (**a**) BSA and its conjugates at different conditions, (**b**) STI and its conjugates at different conditions. Concentration of both native proteins and corresponding conjugates was 1 mg/mL; concentration of DMSO and DMF after addition to the protein solution was 0.4%.

BSA is a protein consisting of 583 amino acids with six α-helices, with 66% α-helicity contributed by its 385 amino acids [34,35]. In our study, the circular dichroism spectrum of native BSA (Figure 4a) has two negative bands at 208–209 and 220–222 nm, which is consistent with previously described data for this protein and completely corresponds to the α-helical structure of BSA [36,37]. When DMSO was added to BSA, the circular dichroism spectrum of this protein remained virtually the same, indicating that the α-helix structure was preserved and peaks at 209 and 220 nm due to n⃗π* and π⃗π* transitions. In contrast to DMSO, the introduction of DMF leads to a change in the CD spectrum. Both BSA_DMF_–BVA and BSA_DMSO_–BVA spectra show the disappearance of characteristic peaks, indicating protein–protein and protein–solvent interactions [38]. In the case of BSA conjugates, protein unfolding most likely occurs as spirals are transformed into disordered structures.

The study of CD spectra of proteins, as well as conjugates obtained using DMF and DMSO, demonstrated differences in their behavior. First, the spectra of native proteins in 10 mM PBS were examined. In the buffer, both proteins showed characteristic shapes and peaks. When DMF was added to both proteins, artifacts were observed, indicating a change in structure (Figure 4a,b, dash lines). Despite the fact that four times less organic solvent was added than in the synthesis method, the circular dichroism spectra in DMF did not turn out any different. At the same time, at such ratios, DMSO did not cause changes, leading to protein aggregation.

The spectra of STI and the corresponding STI conjugates were recorded in the same nm range to assess the state of the native protein and conjugates. Thus, the spectrum of the native protein showed that its structure lacks α-helices, and the structure is represented by β-structures, which is consistent with the previously described data for STI [39]. The introduction of DMSO leads to a slight shift in the position of the negative peak to the left, indicating an interaction with the solvent (Figure 4b). At the same time, the presence of the same amount of DMF does not allow for obtaining a full spectrum, which clearly demonstrates the differences in the effect of solvents on the protein in solution. Similar dependencies were described above for BSA. As can be seen from Figure 4b, the CD spectrum of the STI_DMF_–BVA conjugate differs from the native protein only in intensity. It should not be forgotten that chemical modification with the formation of new amide bonds took place during the production of hapten–protein conjugates. Therefore, the loss of the secondary structure in the conjugate STI_DMSO_–BVA is most likely associated with the unwinding of the protein molecule and the formation of a more rigid framework during the interaction of free amino groups of the protein with the carboxyl groups of the hapten.

### 3.3. Preparation and Characterization of Rabbit Antisera

**Figure 5:**
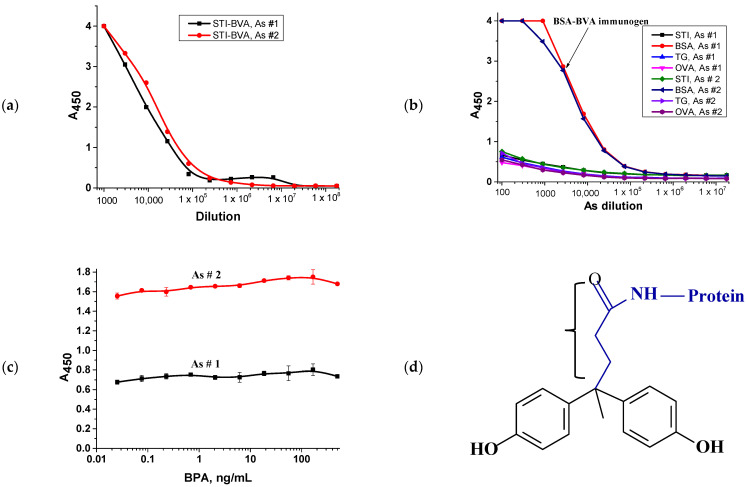
Characteristics of rAs preparations obtained by immunization with the conjugate BSA_DMF_-BVA. (**a**) Titration curves of antisera on the STI_DMF_–BVA conjugate immobilized in microplate wells, (**b**) titration curves of antisera on preparations of non-conjugated proteins, (**c**) competitive curves for the determination of bisphenol A, (**d**) schematic structure of protein–BVA conjugate. The bracket indicates a spacer fragment.

Rabbit antisera (rAs) were obtained by rabbit immunization with the BSA_DMF_–BVA conjugate according to the immunization schedule. The initial titration of the obtained antisera (№1 and №2) was carried out by immobilizing the STI_DMF_–BVA conjugate at a concentration of 3 μg/mL on the solid phase. Titration of the antisera allowed the selection of a dilution corresponding to an optical density at 450 nm of about 1.0 for further competitive interaction (Figure 5a). This dilution was 1:25,000 for both antisera. To assess the specificity of the interaction, a non-competitive interaction was carried out, in which native proteins were immobilized into the microplate wells instead of the STI_DMF_–BVA conjugate. As expected, a strong binding was observed with the BSA protein since it was the carrier protein of the immunogen (Figure 5b). This is why we further immobilized the STI–BVA conjugate since the antiserum was obtained for the BSA–BVA conjugate and a significant number of antibodies in it are directed to binding to BSA (the carrier protein) to avoid cross-reactions. However, at the end of the experiment, it was found that the combination of immunopreparations used in the enzyme-linked immunosorbent interaction demonstrated a lack of competition (Figure 5c). The experiment was carried out several times, but the results were reproduced as expected.

The only logical explanation for this phenomenon would be the assumption that specific antibodies recognize the spacer between the core of the hapten molecule and the protein part (Figure 5d) but do not recognize phenyl radicals. This happens when part of a molecule is shielded, for example, by a portion of a protein, or a spacer influence is observed [40,41]. However, previous studies on the synthesis of the hapten–protein conjugate did not confirm this assumption in any way [42,43]. Moreover, work has been described using a similar synthesis of a conjugate from BVA using the carbodiimide method and subsequent production of monoclonal [14] and polyclonal [44] antibodies to bisphenol A.

Therefore, the next step was to replace the conjugate on the solid phase obtained by replacing the solvent during synthesis.

### 3.4. Evaluation of the Interaction of Antisera with Conjugate Using a Modified Method

The resulting BSA_DMSO_–BVA and STI_DMSO_–BVA conjugates were used to evaluate the interaction of the resulting antisera in noncompetitive and competitive formats.

Interaction of diluted antisera №1 and №2 with STI_DMSO_–BVA or BSA_DMSO_–BVA conjugates immobilized on the solid phase demonstrated the presence of binding in both cases. However, as in the first case, the antisera bound well to the BSA_DMSO_–BVA conjugate due to the fact that it is an immunogen protein. There was a lack of competitive interaction.

**Figure 6:**
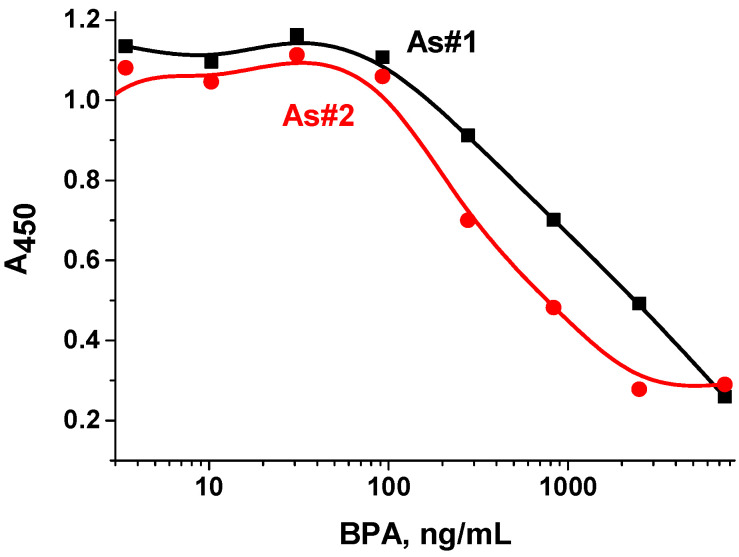
Competitive curves for the determination of bisphenol A using the immobilized conjugate STI_DMSO_–BVA and antiserum №1 (black line) and №2 (red line) obtained by immunizing rabbits with the conjugate BSA_DMF_–BVA.

The introduction of the competitor bisphenol A into the system with immobilized STI_DMSO_-BVA demonstrated the presence of recognition, and competitive interaction curves were obtained for both antisera preparations (Figure 6). It is worth noting the characteristic competitive shape of the curve, as well as adequate optical density and background values. The detection limit of bisphenol A was 61.5 ng/mL for antiserum №2 and 101 ng/mL for antiserum №1. The working ranges were 110–795 ng/mL and 260–5500 ng/mL for the antiserum №2 and №1, respectively. Thus, competitive interaction for the binding sites of specific antibodies turned out to be possible when changing the protocol of conjugate synthesis. The optimal combination of immune reagents was antisera obtained by immunizing rabbits with the conjugate BSA_DMF_–BVA, and STI_DMSO_–BVA used for immobilization into the wells of a microplate.

### 3.5. ELISA Optimization

Based on the obtained primary data, the interaction of the obtained polyclonal antibodies with the analyte was optimized under conditions of competition with the immobilized hapten–protein conjugate. Since the reagent combinations were selected at the previous stage of the work, when the presence of competitive interaction was assessed, the task of this stage was to determine the capabilities of the immunoassay and the achievable values of the detection limit. In addition, the width of the working range of the determined concentrations of bisphenol A and the average statistical deviations in its linear section (IC_20_-IC_80_) were estimated. For this purpose, the concentration of the immobilized hapten–protein conjugate, the dilution of the antisera, and the concentrations of bisphenol A were varied with the first two parameters selected.

Titration of the antiserum in the absence of a competitor showed that the maximum optical density was observed at a STI_DMSO_–BVA concentration of 3 μg/mL. The dilution of antiserum №1 corresponding to an optical density of 1.0 was 1:100,000, 1:60,000, 1:32,000, 1:22,000 for 3, 2, 1, and 0.5 μg/mL of STI_DMSO_–BVA. For antiserum №2, these dilutions were 1:200,000, 1:120,000, 1:64,000, and 1:40,000, respectively.

**Figure 7:**
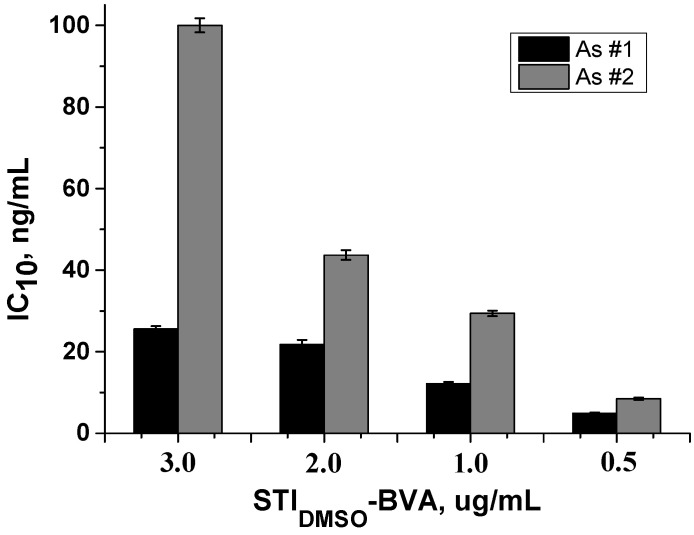
Histogram showing the change in the IC_10_ parameter with a change in the concentration of the immobilized conjugate STI_DMSO_–BVA. rAs dilution was 1:25,000.

Also, using the selected concentrations of the sorbed hapten–protein conjugate and the selected dilution of the antiserum, the curves for determining bisphenol A in the buffer were obtained. Figure 7 shows a histogram demonstrating the change in the IC_10_ value with an increase in the concentration of the sorbed hapten–protein conjugate at the selected dilution of the antiserum. It is evident that the minimum values were achieved with the immobilization of 0.5 μg/mL of STI_DMSO_–BVA.

Additionally, the values of average deviations in the working ranges were estimated. Thus, for antiserum №1, the ranges of deviation values in the working ranges were 2.5–6.5%, 0.2–3.5%, 0.5–4.4% and 0.16–2.3% for 0.5, 1, 2 and 3 μg/mL of STI_DMSO_–BVA. For antiserum №2, these ranges were 9.6–17.1%, 1.7–7.1%, 2.2–6.8%, and 4.1–10.3%, respectively.

Despite the fact that the lowest detection limit was observed at 0.5 μg/mL, the concentration of 1.0 μg/mL conjugate was chosen as the optimal one since the values of standard deviations in the working range are significantly lower compared to the corresponding data at 0.5 μg/mL. An increase in the concentration of the immobilized conjugate leads to a shift of the curve to the region of high concentrations and an increase in the IC_10_ value for bisphenol A.

**Figure 8:**
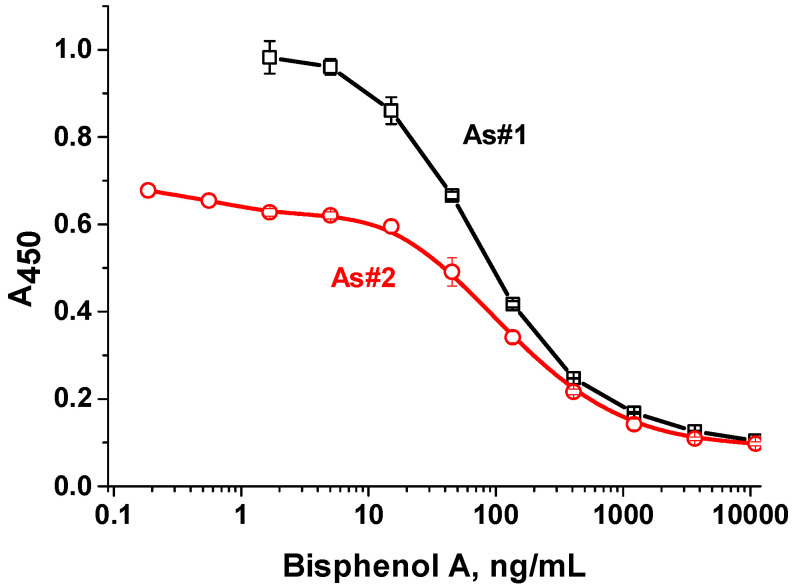
Calibration curves for bisphenol A detection (*n* = 3) for rAs №1 (black line) and №2 (red line).

Under the selected conditions, calibration curves for the determination of bisphenol A were obtained (Figure 8). The detection limit of bisphenol A was 8.3 ng/mL, and the working range was 18.5–290.3 ng/mL for antiserum №1. For rAs №2, similar analytical parameters were found for 14.7 ng/mL and 31.6–430.0 ng/mL, respectively.

It should be noted that the proposed approach expands the scope of application of the concept of heterologous immunoassay in detecting haptens. It was confirmed by a number of studies that the use of an immunogen and a hapten–protein conjugate with different modifications of hapten for competitive immunoassay increases the selectivity of analysis and decreases the detection limits [45,46,47,48,49,50,51]. This effect is explained by the fact, that subpopulations of polyclonal antibodies simultaneously recognize structural elements of the native hapten, its regions modified for conjugation, and the binding site with the carrier protein in the immunogen but not the hapten itself. The functional groups of immunogens are excluded from signal generation in the competitive assay because the immobilized hapten–protein conjugate is obtained by a different technique and does not contain the same additional structures. The advantages of heterologous immunoassay can also be realized in cases where the immunogen and the competing hapten–protein conjugate are obtained using the same binding reaction but in different reaction media, as shown in the present work.

### 3.6. Water Samples Analysis

Fortified water samples were analyzed under optimal conditions selected for the determination of bisphenol A in buffer. Since a low molarity buffer (10 mM) was used in this work, it helped to avoid additional manipulations with drinking water. Thus, known concentrations of the analyte were added to samples of drinking water that did not contain bisphenol A, and the samples were added at the stage of competitive interaction without additional preparation. The only change in the composition of the medium that was carried out was an increase in the concentration of the Tween-80 surfactant in PBST from 0.05% to 0.1%. This buffer was used to prepare antiserum solutions at the stage of water analysis, to which nothing else was added. Since the working ranges for the two antisera overlap, analyte concentrations that fall within the sample range were taken. Thus, the samples analyzed in this way demonstrated adequate levels of bisphenol A detection—from 78.5 to 114.7% (Table 1). The obtained results demonstrate the potential in terms of sample analysis and also confirm the possibility of determining bisphenol A using the obtained antisera and the selected combination of reagents (primarily the STI-BVA conjugate obtained using DMSO).

### 3.7. Possible Explanation of the Observed Differences and Its Confirmation

Despite the fact that both DMSO and DMF solvents are widely used in organic synthesis and are used to obtain hapten–protein conjugates, a possible explanation may lie in their inertness towards the substances involved in the synthesis process [52]. The search for a source in the literature that would explain why a particular solvent should be used in the preparation of hapten–protein conjugates was not successful. As a rule, the authors indicate that such a technique was used during the synthesis process, with or without modifications. The search led to fundamental scientific problems related to the behavior of these solvent molecules. Molecules of both solvents are capable of forming clusters that promote electron delocalization. Despite the study of the behavior of the solvents themselves in different environments, as well as a thorough study of the products of the chemical reaction, it is obvious that a single answer to this question has not been obtained to date, since some of the radicals and intermediate products cannot be identified [52].

In this work, we do not pretend to have absolute knowledge; we only compare the facts known to date with the results of testing immune preparations. Both aprotic solvents with high dipole moments are capable of forming complexes with water molecules, but the interaction energy of DMSO with water molecules is higher than in the case of DMF [53]. DMSO and DMF are very similar in structure, but in the latter, two methyl groups are linked to electron-rich nitrogen. In addition, the formation of a good leaving group in transesterification reactions contributes to a higher yield and degree of modification [54].

Organic reactions provided in DMSO [55] showed higher stability of water–DMSO complexes compared to DMF. However, having studied the literature, there is most likely a reduced yield of the reaction product when using DMF compared to conjugates obtained using DMSO as a solvent [54,56,57]. Satellite products identified by chromatographic methods include products of incomplete substitution and reaction, acylated peptides, lactams and others [58]. Mixtures of more polar solvents such as DMSO do not produce such reaction byproducts. There may be several reasons for the lack of recognition. Some differences observed in the FT-IR spectra for conjugates synthesized using DMF did not reveal critical differences from the other pairs of conjugates.

The CD spectra clearly show that DMF and DMSO have different effects on the behavior of native proteins in solution (Figure 4). It is possible when DMF is introduced into the protein solution, the protein changes its initial conformation and folds the amino groups inward, making them inaccessible for modification in the STI molecule. After dialysis, the protein unfolds again, but the existing groups remain unmodified. Therefore, in the case of DMF, the structure after modification is partially preserved in the STI–BVA conjugate after dialysis as a result of an incomplete reaction.

**Figure 9:**
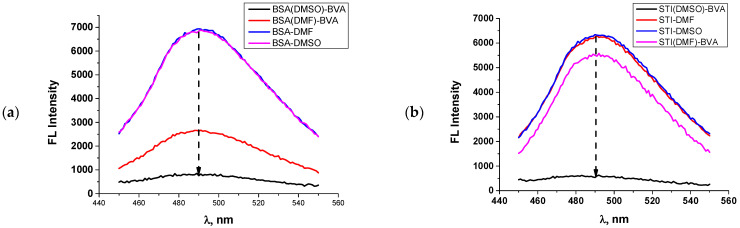
Dependence of fluorescence intensity of fluorescamine-labeled protein preparations and conjugates obtained using DMF and DMSO as solvents. (**a**) Dependencies for BSA and corresponding conjugates. (**b**) Fluorescence spectra for STI preparations and corresponding conjugates. The dash line indicates the position of 490 nm wavelength.

The assumption about the different availability of amine groups in the course of protein modification in different media is based on our experimental results obtained in experiments of these groups testing for their coupling with fluorescamine [19]. The number of surface amino groups for BSA is known and depends on pH, as shown in the classic work of Habeeb. Thus, there are about 30 accessible amino groups from lysine residues on the BSA surface at pH 7.4. In total, BSA contains 59 lysine residues and one N-terminal amino group, but some of them are hidden inside the globule [21]. The implemented study of BSA and its conjugates showed that the fluorescamine-caused fluorescence of the original protein does not depend on the type of added solvent—DMF or DMSO at equal concentrations (0.4% before dilution at initial (1 mg/mL) protein solution, and 0.008% after 50-fold dilution (Figure 9). The initial protein solutions (BSA and STI) were mixed with DMSO and DMF in the same ratio and concentration as in the synthesis of hapten–protein conjugates. This was done to simulate the conjugation conditions and to evaluate the fluorescence of the labeled proteins.

However, when using the BSA_DMF_–BVA conjugate synthesis technique, a fluorescamine conjugate is formed with 12 available amino acids compared to 30 in the original protein (Figure 9a, Table 2). At the same time, the BSA_DMSO_–BVA conjugate is formed in which only three available amino acids are available when using DMSO as a solvent. This means that the reaction yield is higher. In the case of SIT, the dependence is similar (Figure 9b, Table 2). The original Kunitz (STI)-type protein contains 11 primary amino groups—10 lysine residues and 1 terminal amino group [22]. When obtaining a conjugate using DMF as a solvent, nine amino groups remain in the protein, and when using DMSO, less than one, i.e., the reaction is complete. This is probably the key factor that ultimately affects the immunogenicity of the preparation and the production of specific antibodies to the target analyte. Several studies demonstrated a small reaction yield (5–10%) when modifying BSA with the studied hapten, as shown in the work of Torres et al. [59]. Thus, the importance of the solvent for the hapten used in the reaction medium is great, and it can dramatically affect the quality of the resulting antibody preparations.

Additionally, the immunization of animals with the BSA_DMSO_–BVA conjugate was provided as a continuation of this work. As expected, higher antibody titers were noted when the STI_DMSO_–BVA (1 µg/mL) was immobilized, as well as the lower detection limit of BPA compared to the antisera obtained against the BSA_DMF_–BVA conjugate. The analytical parameters obtained for the new antisera are summarized in Table 2. They were obtained on the basis of the corresponding calibration curves as the dependence of optical density on the concentration of BPA. As seen from Table 2, the higher hapten loading on the BSA surface obtained by conjugation in the presence of DMSO contributed to the production of antibodies with a higher titer (Table 3). The values 1:340,000 and 1:820,000 were obtained for antisera against BSA_DMSO_–BVA compared to 1:32,000 and 1:64,000 for the antisera obtained for BSA_DMF_–BVA. The limits of BPA detection were 6.3, 44.7, and 25.0 ng/mL when using antisera collected on the 35th, 49th, and 63rd day of immunization by BSA_DMSO_–BVA.

Thus, the above data confirmed the importance of choosing an organic solvent. The analysis of conjugates on the available amino groups showed that when using DMSO as an organic solvent, conjugates with a higher load are formed. In this case, the reaction occurs almost completely compared to DMF in which free amino groups remain (Table 2). The study of conjugates and native proteins by circular dichroism spectroscopy showed that the differences in the behavior of proteins in the presence of an organic solvent are determined by their differences in primary and secondary structures. Thus, the spectrum of the native protein showed that its structure lacks α-helices, and the structure is represented by β-structures, which is consistent with the previously described data for STI [39]. As for BSA, it is a protein that has six α-helices, and most of the amino acids are involved in the α-helical structure [36,37]. Since various bonds (electrostatic, van der Waals, hydrogen) are formed during the formation of the secondary and tertiary structure, interference with these interactions can cause deviations from the initial state of the protein to a partially or completely inactive one, ultimately affecting the stability of the protein globule and the availability of its functional groups.

## 4. Conclusions

A comparison of hapten–protein conjugates obtained using DMF and DMSO showed that the solvent used in the synthesis process can dramatically affect the results of the study. Thus, the results obtained when replacing DMF with DMSO in the synthesis procedure and subsequent recognition of the target analyte turned out to be interesting. The optimal combination of reagents for interaction in the ELISA system turned out to be an antibody to BSA_DMF_–BVA, and STI_DMSO_–BVA immobilized in the solid phase. The results of optimization of the conditions for conducting analyses and testing prepared water samples demonstrated the efficiency of the approach used. In addition, the developed protocols and tests may be promising from the point of view of environmental monitoring and the development of other analytical systems requiring the production of conjugates and specific antibodies. The advantages of heterologous immunoassay can also be realized in cases where the immunogen and the competing hapten–protein conjugate are obtained using the same binding reaction, but in different reaction media, as shown in the present work. It is planned to continue work in the future in order to understand more about the effect of the solvent on obtaining the final product.

## Figures and Tables

**Table 1:** Results of testing fortified water samples using two antisera.

Bisphenol A, ng/mL	rAs №1		rAs №2	
	Founded, ng/mL	Founded, %	Founded, ng/mL	Founded, %
50	54.7	109.4	44.5	89.0
130	102.0	78.5	133.0	102.3
200	185.6	92.8	229.4	114.7

**Table 2:** Fluorescence values and the corresponding numbers of amino groups for each conjugate and the original protein.

Parameter	BSA_DMF_–BVA	BSA–DMF	BSA_DMSO_–BVA	BSA–DMSO
Fluorescence intensity	2632	6903	758	6838
Number of free amino groups	12	30	3	30
	**STI_DMF_–BVA**	**STI–DMF**	**STI_DMSO_–BVA**	**STI–DMSO**
Fluorescence intensity	5503	6262	570	6313
Number of free amino groups	9	11	1	11

**Table 3:** Results of testing antisera obtained under immunization of rabbits with the BSA_DMSO_-BVA.

Immunization with BSA_DMSO_–BVA, Day of Blood Collection	Titer in Non-Competitive ELISA	Titer in Competitive ELISA	IC_10_, ng/ml	IC_20_, ng/ml	IC_80_, ng/ml
35th	1: 340,000	1: 170,000	6.3	31.9	827
49th	1: 820,000	1: 410,000	44.7	113.9	2797
63rd	1: 820,000	1: 410,000	25.0	75.0	3200

## Data Availability

The original contributions presented in the study are included in the article, further inquiries can be directed to the corresponding author.
